# Viral immune evasins impact antigen presentation by allele-specific trapping of MHC I at the peptide-loading complex

**DOI:** 10.1038/s41598-022-05000-9

**Published:** 2022-01-27

**Authors:** Sunesh Sethumadhavan, Marie Barth, Robbert M. Spaapen, Carla Schmidt, Simon Trowitzsch, Robert Tampé

**Affiliations:** 1 Institute of Biochemistry, Biocenter, Goethe University Frankfurt, Max-von-Laue-Str. 9, 60438 Frankfurt/Main, Germany; 2 Interdisciplinary Research Center HALOmem, Charles Tanford Protein Center, Institute of Biochemistry and Biotechnology, Martin Luther University Halle-Wittenberg, Kurt-Mothes-Str. 3a, 06120 Halle, Germany; 3 Department of Immunopathology, Sanquin Research, Plesmanlaan 125, 1066 CX Amsterdam, The Netherlands

**Keywords:** Isolation, separation and purification, Mass spectrometry, Immunochemistry, Antigen processing and presentation, Immune evasion, Biochemistry, Biological techniques, Cell biology, Immunology

## Abstract

Major histocompatibility complex class I (MHC I) molecules present antigenic peptides to cytotoxic T cells to eliminate infected or cancerous cells. The transporter associated with antigen processing (TAP) shuttles proteasomally generated peptides into the ER for MHC I loading. As central part of the peptide-loading complex (PLC), TAP is targeted by viral factors, which inhibit peptide supply and thereby impact MHC I-mediated immune responses. However, it is still poorly understood how antigen presentation via different MHC I allotypes is affected by TAP inhibition. Here, we show that conditional expression of herpes simplex viral ICP47 suppresses surface presentation of HLA-A and HLA-C, but not of HLA-B, while the human cytomegaloviral US6 reduces surface levels of all MHC I allotypes. This marked difference in HLA-B antigen presentation is echoed by an enrichment of HLA-B allomorphs at US6-arrested PLC in comparison to ICP47-PLC. Although both viral factors prevent TAP-mediated peptide supply, our data imply that MHC I allomorphs favor different conformationally arrested states of the PLC, leading to differential downregulation of MHC I surface presentation. These findings will help understand MHC I biology in general and will even advance the targeted treatment of infections depending on patients’ allotypes.

## Introduction

Major histocompatibility complex class I (MHC I)-mediated presentation of antigenic peptides to cytotoxic T-lymphocytes (CTLs) is fundamental in mounting an immune response against virally infected or malignantly transformed cells^1,2^. In nucleated cells, presentation of antigenic peptides is initiated by the transport of proteasomal degradation products from the cytosol into the lumen of the endoplasmic reticulum (ER)^3^. The process of peptide transport and loading onto MHC I molecules is orchestrated by a dynamic multiprotein translocation and quality control machinery called the peptide-loading complex (PLC)^2,4^. Central to the PLC is the heterodimeric ATP-binding cassette (ABC) transporter TAP, which powers translocation of cytosolic peptides into the ER lumen by ATP binding and hydrolysis^5–7^. The other PLC constituents, the oxidoreductase ERp57, the lectin-like chaperone calreticulin, and the MHC I chaperone tapasin, play key roles in stabilizing peptide-receptive MHC I heavy chain (hc)/β_2_-microglobulin (β_2_m) heterodimers. A fraction of transported peptides is processed by ER-resident aminopeptidases 1 and 2 (ERAP1/2) prior to loading onto MHC I molecules^8,9^. Tapasin and the second-stage MHC I chaperone TAP-binding protein related (TAPBPR), which primarily acts in the ER-Golgi intermediate (ERGIC), edit the MHC I peptidome for high-affinity binding of peptides^4,10^. Stable peptide-MHC I (pMHC I) complexes can exit the ER and travel via the secretory pathway to the cell surface where they present their antigenic cargo to CTLs. MHC I molecules are broadly classified into classical (HLA-A, HLA-B, and HLA-C) and non-classical alleles (HLA-E, HLA-F, HLA-G, MR1, and CD1), both of which regulate and modulate the immune response in different ways, especially during chronic viral infections^11–16^. Classical MHC I molecules are highly polymorphic with 4233 HLA-A, 5240 HLA-B, and 3953 HLA-C allotypes described to date after accounting for the synonymous mutations (http://hla.alleles.org/nomenclature/stats.html, accessed Dec 2021).

Due to its central role in an adaptive immune response, TAP is the prime target of many DNA viruses and frequently down-regulated in tumors^17–22^. During virus-host coevolution, different genera of herpesviruses independently of each other acquired highly efficient ways to block TAP-mediated peptide transport^23^. Upon TAP inhibition, empty MHC I molecules are retained in the ER lumen and are consequently unable to display antigens at the cell surface to the immune system^18,19,23–28^. Remarkably, the viral inhibitors identified so far lack structural homology, bind to different regions, and arrest distinct conformations of the TAP complex^17,19,29^. Common to all TAP inhibitors is that they arrest the transporter in a transport-incompetent state, thereby blocking peptide supply to the ER lumen. This shortage of peptides, in turn, leads to a reduction of peptide-loaded MHC I molecules on the cell surface and helps the virus escape the host immune response^17,20,21,30^.

The immediate early ICP47 of Herpes simplex virus (HSV1) blocks translocation of peptides by wedging into the peptide-binding cleft of the TAP transporter without affecting ATP binding^31–35^. In contrast, the unique short sequence 6 (US6) encoded by human cytomegalovirus (HCMV) is an ER-resident type-I transmembrane glycoprotein with an active ER-lumenal domain interacting with human TAP, which inhibits TAP by a distinct mechanism from ICP47^36–42^. Infection with HSV2, particularly in the presence of ICP47, was shown to specifically down-regulate HLA-C surface presentation in human lymphoblastoid cell lines^13^. US6 was shown not to inhibit cell-surface expression of TAP-independent HLA-A*02:01 but to  reduce the cell-surface expression of HLA-B*27:05 (ref.^43^). However, the mechanism by which such viral factors affect the different MHC I allomorphs has not been addressed so far. We therefore conducted a comprehensive analysis of the cellular and biochemical consequences of ICP47 and US6 expression on PLC composition. Expression of the immune evasins during viral infection was mimicked by a tightly regulated conditional transcription of the viral genes in different stably transduced cell lines. We uncovered a differential cell surface presentation of MHC I allomorphs upon TAP inhibition by ICP47 or US6, which were consistent with the alterations in the MHC I composition in the PLC arrested by ICP47 or US6. Our results reveal a molecular mechanism behind allele-specific blockage of MHC I antigen presentation and provide opportunities for the development of new treatments for infectious diseases.

## Results

### Stable cell lines for the conditional expression of ICP47 or US6

**Figure 1:**
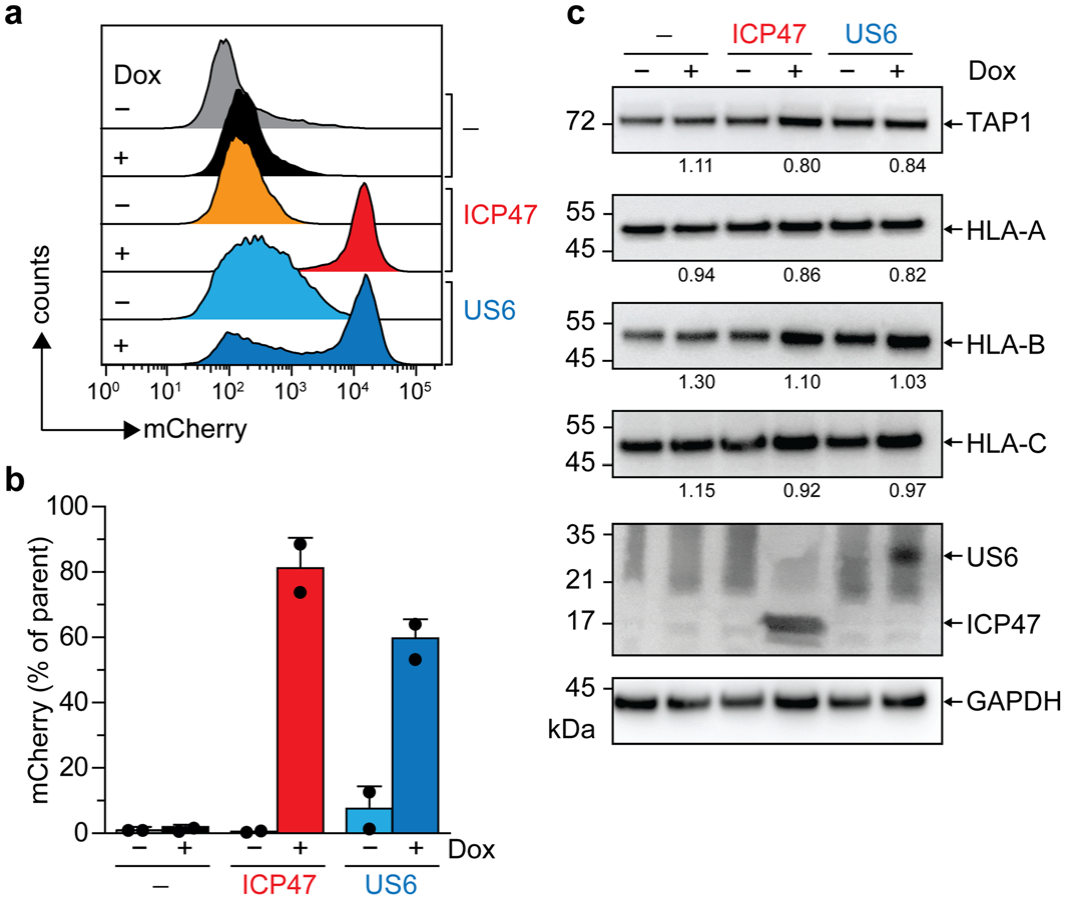
Conditional expression of ICP47 and US6 in Raji cells. (**a**) Raji cells were stably transduced with ICP47- or US6-encoding lentivirus. The expression of ICP47 and US6 was induced with 2 µg ml^−1^ doxycycline (Dox) for 16 h. Protein expression was monitored by bicystronic mCherry expression via flow cytometry. The histogram shows mCherry expression in ICP47- (orange/red) and US6-encoding (cyan/blue) Raji cells and wild-type Raji cells (grey/black) upon doxycycline induction. (**b**) Quantification of mCherry expression in ICP47 (orange/red bar) and US6 (cyan/blue bar) expressing cells in comparison to the non-induced (grey/black bar) cells (n = 2, mean ± SD). (**c**) Immunoblot analysis of TAP1, HLA-A, HLA-B, HLA-C, and SBP-tag in total cell lysates of stably transduced non-induced or induced Raji cells (ICP47 or US6 transduced) or wild-type Raji cells (–). GAPDH was used as a loading control. The quantification of densitometries of TAP1, HLA-A, HLA-B, and HLA-C normalized to the loading control GAPDH in the whole cell lysate of doxycycline-induced cells compared to non-induced cells is depicted below the immunoblots.

To compare the effects of the immune evasion factors on MHC I surface presentation, we generated stably transduced Raji and Mel JuSo cell lines for conditional expression of ICP47 or US6 using a doxycycline-inducible expression system^44^. Both viral inhibitors were equipped with a C-terminal SBP-tag for affinity purification, followed by an internal ribosomal entry site (IRES2) driving bicistronic expression of mCherry as a fluorescent reporter. Upon induction with doxycycline, expression of the viral factors was monitored by flow cytometry using mCherry and by immunoblotting of the cell lysates against the SBP-tag of each viral factor (Fig. 1). Upon doxycycline induction, bicistronic expression of mCherry was observed in > 65% of Raji cells (Fig. 1a,b) and > 75% of Mel JuSo cells (Supplementary Fig. 1a,b) in comparison to the non-induced cells. To establish homogenous cell populations, we generated monoclonal cell lines by subjecting the transduced Raji or Mel JuSo cells to fluorescence-activated cell sorting (FACS) gated on doxycycline-induced mCherry expression (Supplementary Fig. 1d). To assess the overall effects of the conditional expression of the viral factors, we compared the protein expression levels of TAP1, HLA-A, HLA-B, and HLA-C in cell lysates derived from non-induced and induced cells by quantitative immunoblotting (Fig. 1c). Upon doxycycline induction, we did not observe a substantial variation in the overall expression of TAP1 and different MHC I allomorphs in either Raji (HLA-A*03:01, HLA-B*15:10, and HLA-C*04:01) (Fig. 1c) or Mel JuSo cells (HLA-A*01:01, HLA-B*08:01, and HLA-Cw7) (Supplementary Fig. 1c), demonstrating that the protein expression levels of central components of the antigen presentation pathway are not altered upon expression of ICP47 or US6. Thus, specific changes in the steady-state levels of the MHC I allomorphs by synthesis and degradation can be excluded.

### Induced expression of ICP47 or US6 blocks TAP-mediated peptide supply into the ER

**Figure 2:**
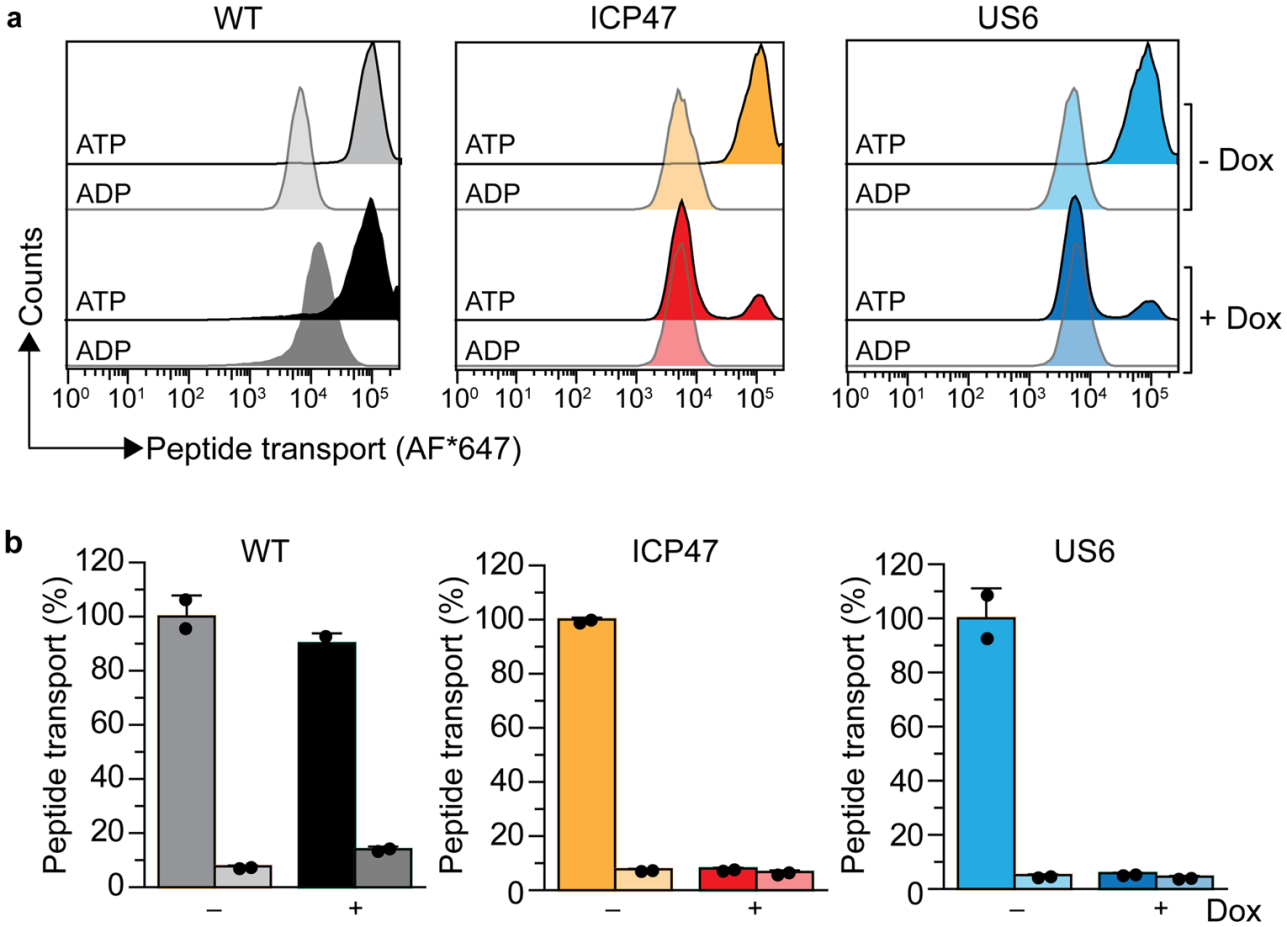
ICP47 and US6 inhibit peptide translocation. (**a**) The non-induced and doxycycline-induced cells were semi-permeabilized with streptolysin O (SLO) and peptide translocation was performed for 15 min at 37 °C with fluorescently labeled (30 nM) NST^AF647^ peptide and ATP or ADP (10 mM each), and analyzed by flow cytometry. Peptide translocation in wild-type cells was unaffected by doxycycline treatment. The non-induced cells translocated peptides, whereas TAP-mediated peptide transport decreased to background levels upon doxycycline (Dox)-induced expression of ICP47 or US6. (**b**) Quantification of peptide translocation as MFI of the Alexa-Fluor^647^ for ATP (dark bar) versus ADP (lighter bar) samples is shown as relative translocation rate (%). The induction of ICP47 and US6 reduced the TAP-dependent peptide translocation to background (n = 2, mean ± SD).

To show that induced expression of ICP47 and US6 blocks peptide translocation into the ER lumen, we performed single-cell-based transport assays^45^. This approach relies on the TAP-dependent transport of fluorescently labeled reporter peptides in semi-permeabilized cells and their subsequent accumulation in the ER lumen^45–47^. We demonstrate that ATP-dependent transport of the RRYQNSTC^AlexaFluor647^L peptide (NST^AF647^) is inhibited in doxycycline-induced cells expressing ICP47 and US6, while the parental and non-induced cells displayed a high level of ATP-stimulated transport (Fig. 2a,b). These data demonstrate that the induced expression of ICP47 or US6 in the monoclonal cell lines prevents TAP-mediated peptide supply into the ER.

### Viral factors alter MHC I surface expression

**Figure 3:**
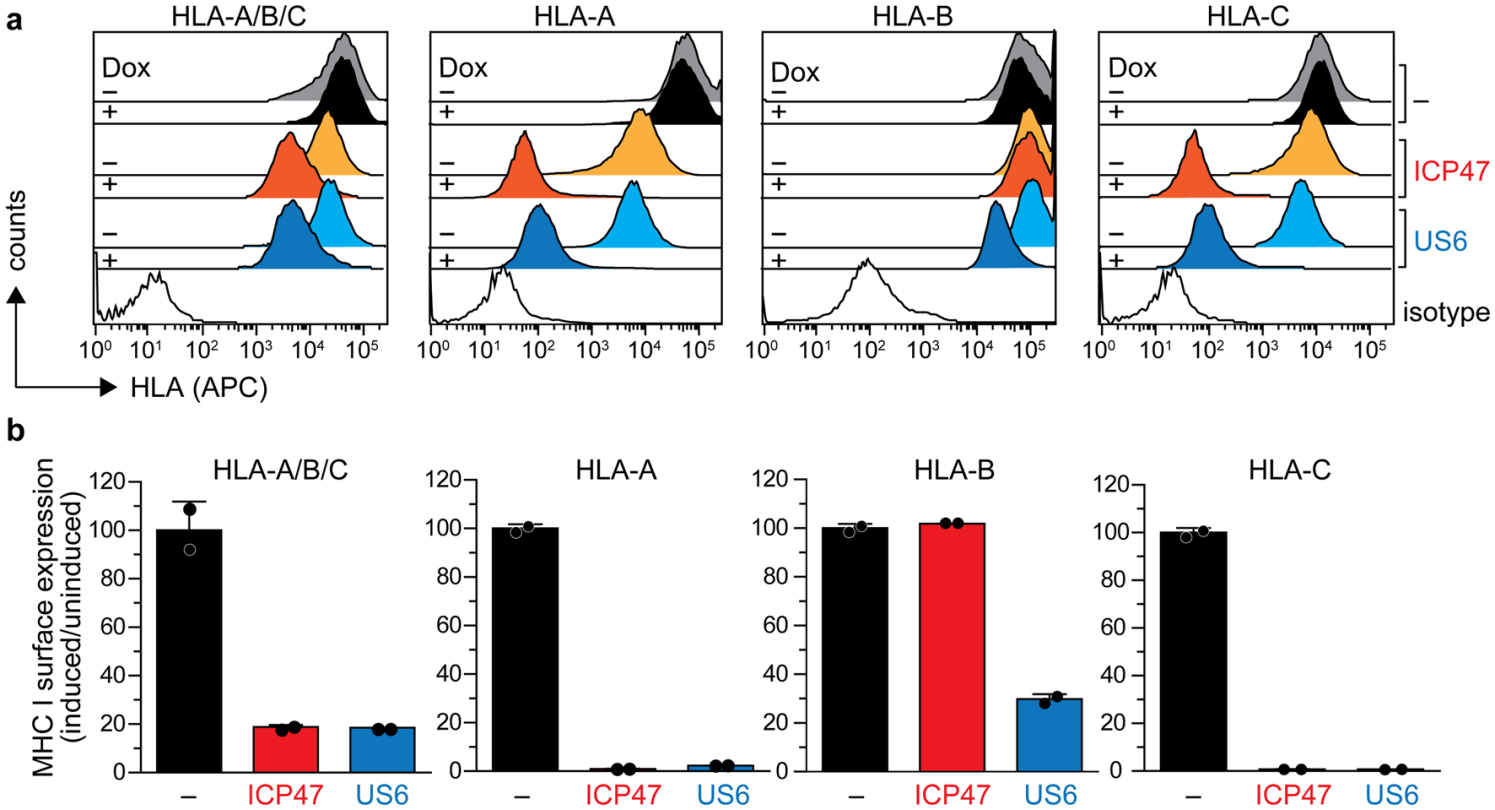
MHC I surface expression is differentially affected by ICP47 and US6. (**a**) Expression of viral factors ICP47 or US6 was induced with doxycycline (Dox) in stably transduced Raji cells. MHC I surface expression was monitored either by direct immunostaining and flow cytometry analysis using allophycocyanin (APC)-conjugated pan-HLA antibody (W6/32) and APC-conjugated HLA-A (A*03:01), or by indirect immunostaining of HLA-B (B*15:10), and HLA-C (C*04:01) with primary antibody followed by APC-conjugated secondary antibody. MHC I surface expression in wild-type (grey/black), ICP47-positive (orange/red), and US6-positive (cyan/blue) Raji cells is displayed as histogram before and after doxycycline induction, respectively. The isotype control is shown in white. (**b**) MHC I surface expression quantified as ratio of the MFI from the induced to the non-induced cells. The ratios of non-transduced cells were set as 100% (n = 2, mean ± SD).

To further understand the effect on MHC I surface expression upon induced expression of viral factors and shortage of peptides for MHC I loading, we investigated the antigen presentation via classical MHC I allomorphs by flow cytometry. Expression of ICP47 and US6 resulted in an overall reduction (> 80%) of MHC I surface presentation in Raji (Fig. 3a) and Mel JuSo cells (Supplementary Fig. 2a) as revealed by the pan-HLA-A/B/C, conformation-specific antibody W6/32. Next, we confirmed the specificity of several anti-HLA antibodies for the allomorphs HLA-A*03:01, HLA-B*15:10, and HLA-C*04:01 expressed in Raji cells (Supplementary Table 1). As a gold-standard for antibody evaluation, we transfected HAP1 HLA-A/B/C knockout (HLA^KO^) cells^48^ with plasmids encoding each HLA allomorph and subsequently monitored the expression by immunostaining and flow cytometry (Supplementary Fig. 3). We observed no cross-reactivity of the allomorph-specific antibodies, neither in immunoblotting nor flow cytometry (Supplementary Fig. 3). In Raji cells, we found a > 95% reduction in surface expression of the HLA-A and HLA-C allomorphs in comparison to non-induced cells (Fig. 3a,b). Similarly, in Mel JuSo cells expressing HLA-A*01:01, HLA-B*08:01, and HLA-Cw7, the HLA-A and HLA-C surface presentation was reduced by ~ 80% and > 90%, respectively, as compared to non-induced cells (Supplementary Fig. 2a,b). Surprisingly, surface presentation of HLA-B was differentially affected by both viral factors. While US6-expressing Raji cells showed a drastic reduction of HLA-B*15:10 surface presentation (> 70%), its surface presentation was hardly affected upon expression of ICP47 (Fig. 3a,b). In Mel JuSo cells, the impact on HLA-B surface presentation was even further amplified, leading to a threefold increase of HLA-B*08:01 upon expression of ICP47 when compared to a 99% reduction upon expression of US6 (Supplementary Fig. 2a,b). It is worth mentioning that doxycycline addition did not change MHC I cell surface presentation, thus excluding indirect or unspecific effects (Fig. 3a,b and Supplementary Fig. 2a,b). Overall, these findings indicate that although expression of viral TAP inhibitors causes an overall reduction of MHC I cell surface presentation by preventing peptide supply, their effects on the individual MHC I allomorphs, in particular HLA-B allomorphs, are highly variable.

### Viral factors differentially impact the MHC I composition of the PLC

**Figure 4:**
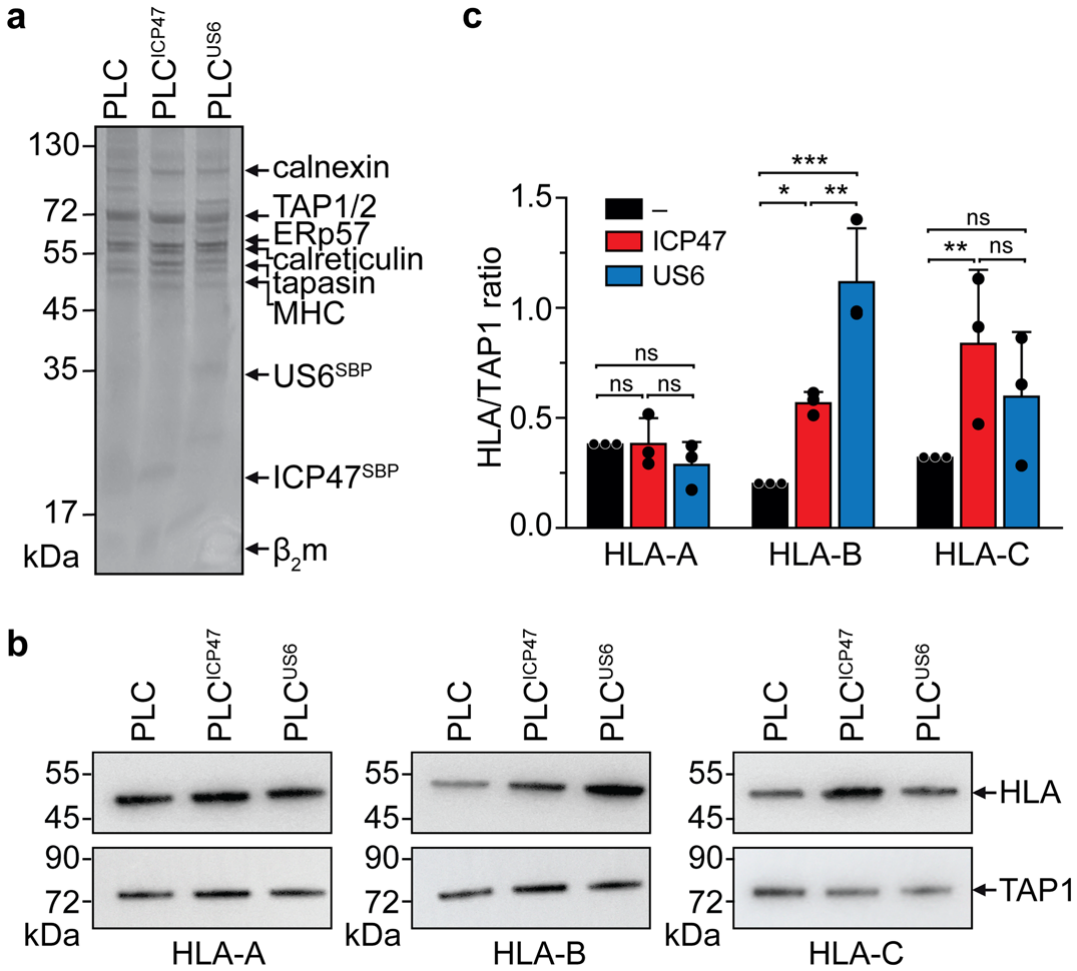
Viral immune evasins differentially change the MHC I repertoire in the PLC. (**a**) PLC was purified via the SBP-tag on ICP47 or US6 (PLC^ICP47^ or PLC^US6^, respectively) after doxycycline induction or with a biotin-conjugated TAP1 antibody (PLC). Affinity-isolated PLCs were further purified by size-exclusion chromatography, and peak fractions were analyzed by SDS-PAGE (10%, Coomassie-stained) and subsequent immunoblotting using HLA-A, HLA-B, HLA-C, and TAP1 specific antibodies (**b**). (**c**) Relative quantification of co-precipitated HLA-A, HLA-B, HLA-C in purified non-inhibited PLC (black bar), ICP47- (red bar), and US6-arrested PLC (blue bar) by densitometry. Intensities of MHC I allomorphs as shown in panel (**b**) were normalized to TAP1 (*P < 0.05, **P < 0.01, ***P < 0.001, n = 3, mean ± SD). Statistical analysis was performed using two-way ANOVA. ns: non-significant.

Raji cells display high expression levels of PLC, which provided us with a key advantage to analyze the assembly of all PLC components in response to viral factor expression. Moreover, Raji cells can be grown in suspension to high density allowing purification of native PLCs in high quality and quantity^49^. To elucidate the effect of ICP47 and US6 expression on the individual MHC I allomorphs, we isolated native ICP47- or US6-arrested PLCs from glyco-diosgenin (GDN)-solubilized Raji cell lysates using the SBP-tag on ICP47 or US6 (Fig. 4). The PLCs were isolated as well-defined monodisperse macromolecular complexes. We then compared the MHC I composition of ICP47- and US6-arrested PLC to that of native PLC, which was isolated via the anti-TAP1 antibody (mAb148.3) and eluted with the specific epitope PADAPE^50^. Native PLCs were subsequently purified by size exclusion chromatography (SEC), and peak fractions were analyzed by SDS-PAGE (Fig. 4a) and immunoblotting against the three different MHC I allomorphs (Fig. 4b). Densitometric analyses revealed a three-fold enrichment of HLA-B in US6-arrested PLC compared to ICP47-PLCs, while the amounts of co-precipitated HLA-A and HLA-C allotypes remained comparable in all purified PLCs (Fig. 4c).

**Figure 5:**
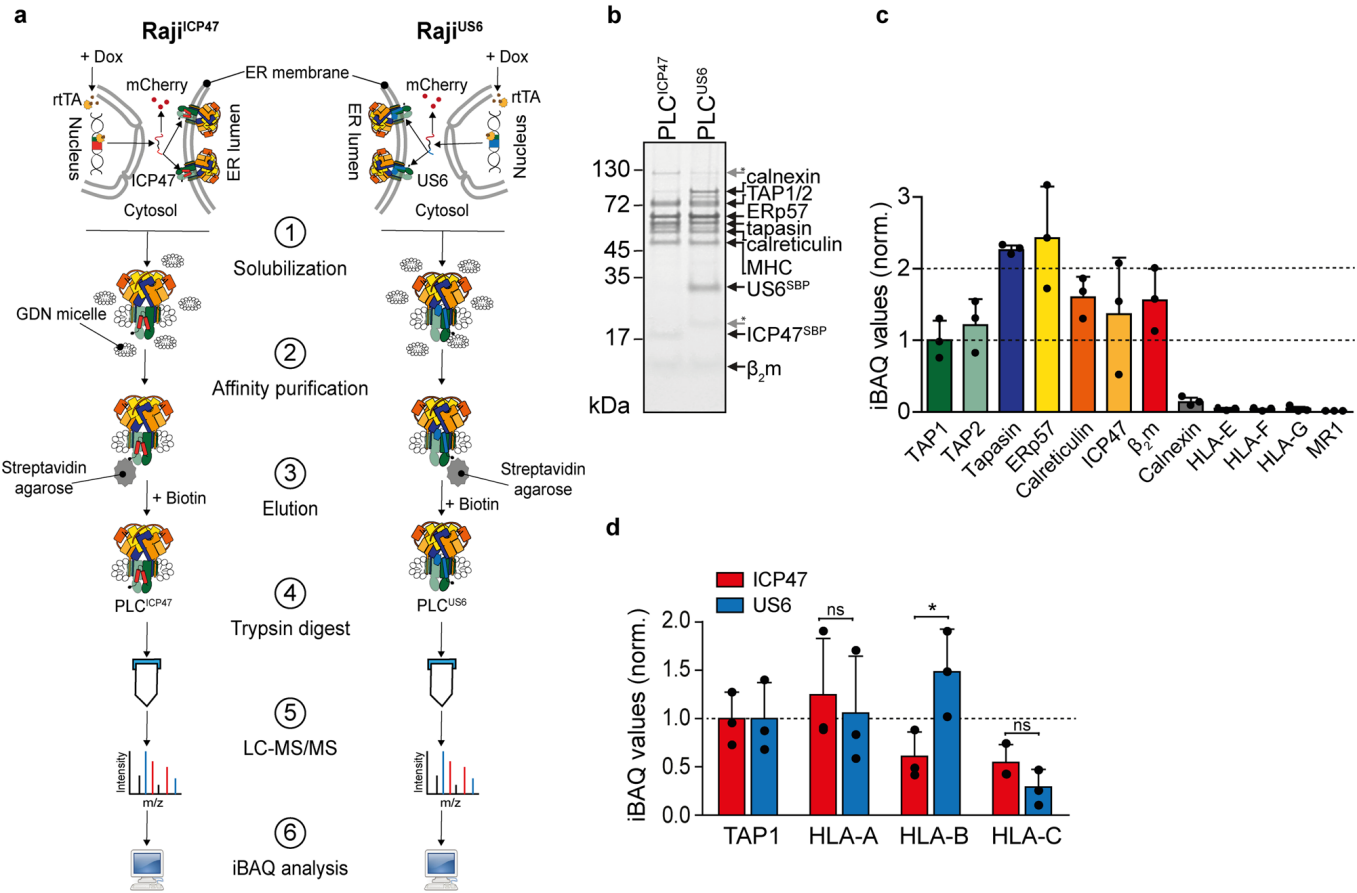
Intensity-based absolute quantification (iBAQ) of the altered MHC I repertoire in ICP47- and US6-arrested PLCs. (**a**) After doxycycline (Dox)-induced expression of ICP47 or US6, the PLC was solubilized by glyco-diosgenin (GDN) and purified via the SBP-tag on ICP47 or US6 (PLC^ICP47^ or PLC^US6^, respectively). Isolated PLCs were hydrolyzed with trypsin, and specific peptides from HLA-A*03:01, HLA-B*15:10, and HLA-C*04:01 were analyzed by mass spectrometry. (**b**) Affinity-isolated PLCs were further purified by size-exclusion chromatography, and peak fractions were analyzed by SDS-PAGE (10%, Coomassie-stained). (**c**) Quantification of all PLC components in the ICP47-PLC and the relative amounts blotted in comparison to TAP1 (n = 3, mean ± SD). (**d**) The quantification of HLA-A*03:01, HLA-B*15:10, and HLA-C*04:01 derived from purified and trypsin-digested ICP47-arrested PLC (red bars) and US6-arrested PLC (blue bars) by iBAQ. HLA-A/B/C iBAQ values were normalized to iBAQ values of TAP1 (*P < 0.05, n = 3, mean ± SD). Statistical analysis was performed using two-way ANOVA. ns: non-significant, rtTA: reverse tetracycline-controlled transcription activator (Tet-On).

To further corroborate the differential effects of viral factors on the MHC I composition in ICP47- and US6-arrested PLCs, stoichiometries in monodisperse complexes were analyzed by label-free quantification mass spectrometry (MS) (Fig. 5a). For this, the purified PLC complexes (Fig. 5b) were hydrolyzed with trypsin, and the obtained peptides were analyzed by liquid chromatography-coupled tandem-MS (LC–MS/MS). Protein intensities were then obtained by label-free quantification using MaxQuant^51,52^. Subsequently, intensity-based absolute quantification (iBAQ)^53^ was used for relative comparison of all PLC components. For this, peptide intensities of each protein were summed and normalized by the number of theoretically observable peptides. PLC components were then normalized in relation to TAP1 in ICP47-PLC, showing a fully assembled PLC (Fig. 5c). iBAQ values of the MHC I allomorphs in ICP47- and US6-arrested PLC confirmed a three-fold enrichment of HLA-B in the US6-arrested PLC compared to the ICP47-inhibited PLC (Fig. 5d). Like the immunoblot analysis, ratios of HLA-A and HLA-C allotypes remained unchanged when ICP47- and US6-arrested PLCs were compared (Fig. 5d). These findings demonstrate that, though both viral factors block TAP and prevent ER peptide supply, ICP47 and US6 have a very different impact on the PLC regarding the recruitment of HLA-B allomorphs.

### Discussion

In this study, we demonstrated that the viral inhibitors ICP47 and US6 alter the MHC I composition of the PLC, thus providing the molecular basis for the different impact on the cell surface expression of specific MHC I allomorphs. To study the effect of viral TAP inhibitors in the absence of potential overlapping factors, we generated monoclonal cell lines, which conditionally express ICP47 or US6 (Fig. 1). Upon doxycycline induction, the expression of ICP47 or US6 completely abrogated TAP-dependent peptide transport (Fig. 2) and led to an overall down-regulation of the MHC I surface presentation (Fig. 3). To our surprise, ICP47 and US6 had different effects on the surface expression of each HLA allotype: in comparison to ICP47 expression, US6 expression caused a three-fold stronger inhibition of HLA-B cell-surface levels (Fig. 3). However, a similarly reduced surface level of both, HLA-C (> 98% inhibition compared to the wild type), and HLA-A (> 96% inhibition compared to the wild type) was observed upon ICP47 and US6 expression (Fig. 3).

Since no global effect on the MHC I expression level was observed in cell lysates in which ICP47- and US6-expressing cells were compared with non-induced cells (Fig. 1c), we rationalized that preferential trapping of MHC I in the ICP47- or US6-arrested PLC was the reason for the different surface levels of HLA allomorphs. As analyzed by co-immunoprecipitation followed by immunoblotting and mass spectrometry, the MHC I repertoire of purified ICP47-PLC and US6-PLC showed a three-fold enrichment of HLA-B in US6-arrested PLC compared to ICP47-arrested PLC. There is no evidence to date that suggests a direct interaction between the TAP inhibitors and MHC I in the PLC. However, such distinct allomorph-specific trapping at the PLC together with a differential reduction in cell surface presentation is consistent with the missing-self hypothesis, which proposes that cells lacking MHC I molecules on their cell surface are targeted by natural killer (NK) cells. HCMV encodes the MHC I mimic UL18 to hide infected cells from NK cells; however, no such molecule has been identified in HSV. Therefore, we argue that HSV needs to allow presentation of HLA-B allomorphs to escape recognition by NK cells. Moreover, the HLA-B allomorphs presented on the cell surface of ICP47-expressing cells likely display an altered peptide repertoire of host origin to evade NK cell recognition –and additionally– inhibit the activation of a CD8^+^ T cell response. ICP47 and US6 are structurally different and vary in their mode of inhibition^35,36,40,49^. ICP47, a soluble protein with a helix-loop-helix structure^54^, targets TAP from the cytosol and competes with peptide binding^31,42^. The active, ER-lumenal domain of US6 allosterically arrests TAP interfering with ATP binding on the cytosolic side of the ER membrane^39,40^. Thus, the inhibition mechanisms of ICP47 and US6 are fundamentally different and these viral factors arrest the TAP complex in different conformations^41,42^. Our data suggest that the different conformational states of the arrested TAP complex results in the altered MHC I allomorph composition in the PLC and is independent of abated peptide supply to the ER. Therefore, we hypothesize that the conformational difference between ICP47- versus US6-arrested TAP results in the preferential recruitment of HLA-B in US6-arrested PLCs compared to ICP47-arrested PLCs. A high-resolution structure of US6-arrested PLC will help understand the detailed mechanism of MHC I allomorph restriction. The present study also indicates that different viral immune evasins have evolved strategies to selectively down-regulate specific HLA allotypes that are actively involved in the surface presentation of viral peptides to CTLs to evade the host immune response. Although we could show the same effect in two unrelated cell lines, a lymphoma (Raji) and a melanoma cell line (Mel JuSo), larger studies need to be conducted to show a general effect with the many different HLA-B allomorphs known to date. Like ICP47 and US6, other virally encoded immune evasins, such as Epstein-Barr virus BNLF2a, cowpox virus CPXV12, and bovine herpes virus UL49.5, vary greatly in terms of structure and topology and have different modes of TAP inhibition^55–60^. Whether down-regulation of MHC I surface expression by these viral inhibitors results from a differential impact on the MHC I repertoire at the PLC needs to be addressed in future studies. In addition, how the down-regulation of specific HLA allomorphs by viral immune evasins modulate the host T-cell response remains an open question.

## Methods

### Constructs

For lentiviral transduction, DNA fragments encoding ICP47, US6, and the streptavidin-binding peptide (SBP) tag were PCR amplified and fused by subsequent overlap PCR to generate ICP47^SBP^ and US6^SBP^ fusion constructs. The final PCR products were cloned into the transfer plasmid pViFCGdBH-IRES2-mCherry^45^ via *Sal*I/*Xba*I restriction sites. The ICP47^SBP^-IRES2-mCherry and US6^SBP^-IRES2-mCherry expression cassettes were PCR amplified and cloned into pCW57.1-MCS1-P2A-MCS2 (Addgene plasmid #71,782)^61^ via *Nhe*I/*Bam*HI restriction sites. The pCW57.1-MCS1-P2A-MCS2 plasmid carries a puromycin resistance gene for selection of stable transfectants and a tetracycline-response element (TRE) promoter for inducible expression. Together with the packaging plasmid SgpΔ2 and the envelope plasmid pMD.G2, pCW57.1-MCS1-P2A-MCS2-ICP47^SBP^ or pCW57.1-MCS1-P2A-MCS2-US6^SBP^ were used for transient transfection of HEK293T cells for lentivirus production. For transient transfection of HAP1 cells, DNA fragments encoding HLA-A*03:01, HLA-B*15:10, and HLA-C*04:01 were generated by custom gene synthesis (GenScript Biotech, USA) and cloned into pcDNA3.1 carrying a hygromycin resistance gene for selection. The ectodomains of HLA*02:01 and HLA-B*44:02 lacking their transmembrane and cytosolic domains were cloned into plasmids pET28 and pET22-b, respectively, and expressed as inclusion bodies in *Escherichia* *coli* Rosetta(DE3)pLysS as described^62^. The construct and inclusion body production of human β_2_-microglobulin (β_2_m) is described^63^. All clones were confirmed by Sanger sequencing.

### Cell lines

All cell lines used in the present study were cultured at 37 °C in a humidified atmosphere with 5% CO_2_. Raji cells (German Collection of Microorganisms, DSMZ no.: ACC 319) and Mel JuSo cells (DSMZ no.: ACC 74) were cultured in RPMI (Gibco) supplemented with 10% tetracycline-negative fetal calf serum (FCS). Raji cells were grown in suspension culture. Cells were seeded at 0.2 × 10^6^ cells ml^−1^, maintained at 1 × 10^6^ cells ml^−1^, and split 1:10 every 72 h. Adherent Mel JuSo cells were seeded at 0.5 × 10^6^ cells in a T-75 cell culture flask and split 1:10 when confluency of 80% was reached.

### Stable monoclonal cells expressing ICP47 or US6 conditionally

Stable cells were prepared by lentiviral transduction. Lentiviral particles were produced in HEK 293 T cells. For the lentiviral particle production, HEK 293 cells were co-transfected with the transfer plasmid (pcW57.1), carrying the gene of interest (GOI) under the control of a Tet-inducible promoter and encoding an IRES2 site followed by the mCherry gene downstream of the GOI, envelope plasmid (pMD.D2), and packaging plasmid (Spg.d2). The lentivirus-containing media supernatant was concentrated with LentiX-concentrator (Takara). The concentrated lentivirus was aliquoted and stored at − 80 °C for long term storage. For transduction of the cells, the lentiviral particles were diluted to a 1.2 ml suspension of 2 × 10^6^ cells with RPMI media without FCS, supplemented with 1 µg DEAE dextrose. The cell suspension was transferred to a 6-well cell culture dish and incubated for 6–8 h. The cells were then supplemented with 1.8 ml of RPMI media supplemented with 10% FCS and incubated for 24 h. The cells were cultured for three passages before stably transduced cells were selected with puromycin, a resistance gene encoded in the transfer plasmid. The cells were diluted to 1 × 10^5^ cells ml^−1^ and supplemented with 3 µg ml^−1^ puromycin. 200 µl of the cell suspension were aliquoted to each well of 96-well cell-culture plates and incubated until the transduced cells grew to confluency. Cells were harvested and induced with 1 µg ml^−1^ doxycycline for 6–8 h. The induced cells were harvested and washed in PBS and resuspended in FACS buffer (1 × DPBS, 2 mM EDTA, 2% FCS). mCherry-positive cells were gated, and single cells were sorted per well in a 96-well cell-culture plate pre-aliquoted with 200 µl conditioned RPMI media with 10% FCS (conditioned media was prepared by the addition of 10% FCS reconstituted fresh RPMI with cell culture supernatant harvested by centrifugation and 0.2-µm sterile filtered in 4:1 ratio). Genomic DNA from monoclonal cells were isolated. Integration sites were amplified by PCR and validated by Sanger sequencing (Microsynth, SeqLab GmbH) to confirm presence of the correct GOI in the genome.

### Protein expression upon doxycycline induction

Raji and Mel JuSo cells were induced with 2 µg ml^−1^ doxycycline for 24 h after the cells reached 70–80% confluency. Mel JuSo cells were harvested by detaching the cells with DPBS supplemented with 5 mM EDTA. After centrifugation at 300 × g for 3 min and washing in ice-cold DPBS, cells were used for further analysis. For large-scale cultures, the Raji cells were adapted to one-liter Erlenmeyer flasks and grown under constant agitation at 125 rpm, 37 °C, and 5% CO_2_. Cells were induced with 2 µg ml^−1^ doxycycline for 48 h before harvesting. Cells were washed in ice-cold PBS and the cell pellet was stored at -80 °C.

### Transfection of HAP1 cells

Wild-type and HLA-A, -B, -C knockout HAP1 cells were cultivated in IMDM media supplemented with 10% FCS^48^. At 70% confluency, the cells were detached by incubation with 5 mM EDTA in 1 × DPBS for 15 min. Cells were harvested by centrifugation at 300 × g for 3 min and washing with 1 × DPBS. 1 × 10^5^ cells were seeded per well of a 6-well plate and supplemented with 3 ml IMDM media. After 24 h, the media was changed and cells were washed with 1 × DPBS, and 1 ml of fresh IMDM media was added. For transfection, 1 µg DNA was mixed per well with 3 µl of Xtreme-GENE transfection reagent (Roche) in 100 µl of OptiMEM media without FCS. The DNA-Xtreme-GENE mix was incubated at room temperature for 20 min. The mixture was added to the cells in a dropwise manner, and the cells were incubated for 48 h. Afterwards, the media was changed to 2 ml complete IMDM media. After 12 h, the transformed cells were selected with 400 µg ml^−1^ of hygromycin for 48 h. After selection of the transformed cells, the media was exchanged to 2 ml of complete IMDM media without antibiotic, and the cells were incubated for 12–14 h before harvesting for immunostaining of HLA.

### Isolation of human PLCs

The harvested cells were thawed on ice and resuspended in solubilization buffer: 20 mM HEPES–NaOH pH 7.4, 150 mM NaCl, 10 mM MgCl_2_, 1% (w/v) protease inhibitor mix, and 2% (w/v) GDN. 5 ml of the solubilization buffer was added per gram of cells. The lysate was homogenized and incubated at 4 °C for 1 h. The lysate was centrifuged at 100,000 ×*g* for 1 h at 4 °C, and the cleared supernatant was incubated with pre-equilibrated streptavidin agarose beads (ThermoFisher, Pierce). After washing the beads three times with washing buffer (20 mM HEPES–NaOH pH 7.4, 150 mM NaCl, 0.02% (w/v) GDN, and 2 mM PMSF), the protein was eluted with 2.5 µM biotin in 20 mM HEPES–NaOH pH 7.4, 150 mM NaCl, and 0.02% (w/v) GDN. The eluate was concentrated with a 100-kDa cut-off concentrator (Amicon-Ultra 0.5 ml, Merck, Millipore). The PLCs were isolated by size exclusion chromatography (Superose 6 3.2/300, GE), and the peak fraction harvested for further analysis.

### Peptide transport assay

0.2 × 10^6^ Raji cells were semi-permeabilized using streptolysin O (2 U ml^−1^) at 4 °C for 15 min and washed to remove residual SLO. Transport was carried out in the presence of 10 mM ATP or ADP, 10 nM NST^A647^ in PBS buffer supplemented with 10 mM MgCl_2_ for 20 min at 37 °C in a 50 µl reaction volume. The transport was stopped by addition of 150 µl PBS supplemented with 20 mM EDTA. The peptide transport was monitored by flow cytometry, and the data were analyzed using FlowJo (TreeStar) software reporting the mean fluorescence intensity.

### Immunoblotting

Protein samples were heated to 95 °C for 10 min with a reducing SDS-sample loading buffer. The samples were analyzed by SDS-PAGE (12%) and subsequent electroblotting onto methanol-activated polyvinylidene fluoride (PVDF) membranes. The blotting membrane was blocked with non-fat milk before incubating with the primary antibody against human TAP1 (mAB148.3), HLA-A/B/C (W6/32), HLA-A (Ab52922), HLA-B (Ab76795), and HLA-C (Ab126722). The immunoblots were analyzed by chemiluminescence using the corresponding secondary antibody and the Clarity Western ECL reagent (BioRad). Chemiluminescence was recorded with a Lumi-Imager (Roche). The signals of the immunoblots were quantified using ImageJ (NIH).

### Flow cytometry

The induction of protein production by doxycycline in stably transduced cells was monitored by flow cytometry (FACS melody, BD Biosciences) using mCherry as reporter. MHC I surface expression of the induced and non-induced cells was monitored with monoclonal antibodies listed in Supplementary Table 1. For surface staining, cells were harvested 14–16 h after induction. Cells were washed twice in ice-cold FACS buffer (1 × DPBS, 2 mM EDTA, 1% BSA). Antibody surface staining procedures were carried out on ice. Cells were first incubated with an FcR-receptor blocking reagent (BioLegend). For direct antibody staining, cells were incubated with an APC-conjugated primary antibody for 30 min in the dark. After washing twice, cells were resuspended in FACS buffer and analyzed by flow cytometry. For indirect staining, cells were stained with an unconjugated primary antibody, washed, and incubated with corresponding APC-conjugated secondary antibody for 20 min. Cells were washed twice and MHC I surface expression was monitored by flow cytometry, and the data were analyzed using FlowJo (TreeStar). The median fluorescent intensities (MFI) were calculated and compared.

### LC–MS/MS sample preparation

50 µg of PLC proteins were precipitated using 1/10 vol. 3 M sodium acetate, pH 5.3 and 3 vol. ice-cold ethanol followed by overnight incubation at − 20 °C. The protein pellet was washed twice with 80% (v/v) ice-cold ethanol and then dried in a vacuum centrifuge. Tryptic digestion in the presence of RapiGest (Waters) was performed according to the manufacturer’s protocol. Briefly, the protein pellet was dissolved in 10 µl 1% (w/v) RapiGest in 25 mM ammonium bicarbonate, pH 8.5, and incubated for 15 min at room temperature. Cysteines were reduced by addition of 10 µl of 50 mM dithiothreitol in 25 mM ammonium bicarbonate, pH 8.5, and by incubation for 30 min at 60 °C. Cysteines were alkylated with 10 µl 100 mM 2-iodoacetamide in 25 mM ammonium bicarbonate, pH 8.5, for 30 min at 37 °C in the dark. Prior to tryptic hydrolysis, the RapiGest concentration was diluted to 0.1% (w/v) with 25 mM ammonium bicarbonate, pH 8.5. For protein digestion, trypsin (Promega) was added at an enzyme-to-protein ratio of 1:20 (w/w) followed by incubation at 37 °C overnight. Subsequently, the RapiGest was degraded by addition of 20 µl of 5% (v/v) trifluoroacetic acid and incubation for 2 h at 37 °C. Degraded RapiGest was removed by centrifugation, and peptides were dried in a vacuum centrifuge.

### LC–MS/MS analysis

Extracted peptides dissolved in 2% (v/v) acetonitrile, 0.1% (v/v) formic acid were separated and analyzed using nano-flow reversed-phase liquid chromatography (DionexUltiMate 3000 RSLCnano System, Thermo Scientific; mobile phase A, 0.1% (v/v) formic acid (FA); mobile phase B, 80% (v/v) acetonitrile (ACN)/0.1% (v/v) FA) coupled with a Q Exactive Plus Hybrid Quadrupole-Orbitrap mass spectrometer (Thermo Scientific). Peptides were loaded onto a trap column (μ-Precolumn C_18_PepMap 100, C_18_, 300 μm I.D., particle size 5 μm; Thermo Scientific), subsequently separated with a flow rate of 300 nl min^−1^ on an analytical C18 capillary column (50 cm, HPLC column Acclaim® PepMap 100, C_18_, 75 μm I.D., particle size 3 μm; Thermo Scientific) with a gradient of 4–90% (v/v) mobile phase B over 92 min and directly eluted into the mass spectrometer. Typical MS conditions were: data-dependent mode; positive ion mode; spray voltage, 2.8 kV; capillary temperature, 275 °C; MS scan range in the Orbitrap, *m/z* 350–1600; MS resolution, 70,000; automatic gain control (AGC) target, 3e6. The twenty most intense ions with charge states of 2 + to7 + were selected for HCD fragmentation, and previously selected ions were specifically excluded for 30 s. MS/MS conditions were: MS/MS resolution, 17,500; fixed first mass, 105.0 m/z; normalized collision energy, 30% and AGC target, 1e5. Internal calibration of the Orbitrap was performed using the lock mass option (lock mass m/z 445.120025)^64^.

### MS data analysis

For protein identification and quantification, raw data were searched against the database of the human PLC complex (see PRIDE 20201029_PLC_ICP47.fasta and 20201029_PLC_US6.fasta) using MaxQuant v1.6.17 (ref.^51,52^) with the following database search settings: enzyme, trypsin; mass accuracy of precursor ions in main search, 4.5 ppm; mass accuracy of fragment ions in main search, 0.5 Da; number of allowed missed cleavages, 2; fixed modifications, carbamidomethylation of cysteine; variable modifications, oxidation of methionine and acetylation of protein N-terminus; quantification, iBAQ; FDR, 1%. For the calculation of protein stoichiometries, relative iBAQ values of three biological replicates were used. For PLC-US6, one replicate was measured in three fractions, which were combined during the MaxQuant search using the fraction parameter. The obtained Protein Groups table was filtered for “potential contaminants”, “only identified by site”, and “reverse”. Relative iBAQ values were calculated as follows: iBAQ/ sum iBAQ.

### Statistical analysis

Statistical analysis was performed by using GraphPad Prism5. For group analysis, two-way ANOVA was used.

## Supplementary Information

Supplementary Information.

## Data Availability

The MS proteomics data have been deposited to the ProteomeXchange Consortium (http://proteomecentral.proteomexchange.org) via the PRIDE partner repository^65^ with the dataset identifier PXD027408.
