# Supplementary material for: Viral immune evasins impact antigen presentation by allele-specific trapping of MHC I at the peptide-loading complex
Source: Sci Rep. 2022 Jan 27;12:1516. doi: 10.1038/s41598-022-05000-9 (PMC8795405; doi:10.1038/s41598-022-05000-9)
Supplement: Supplementary file 1 — Supplementary Information. [file 41598_2022_5000_MOESM1_ESM.pdf]

Supplementary Information

Viral immune evasins impact antigen presentation by allele-specific trapping of MHC I at the peptide-loading complex

Sunesh Sethumadhavan[1], Marie Barth[2], Robbert M. Spaapen[3], Carla Schmidt[2], Simon Trowitzsch[1], Robert Tampé[1,*]

[1] Institute of Biochemistry, Biocenter, Goethe University Frankfurt, Max-von-Laue-Str. 9, 60438 Frankfurt/Main, Germany.

[2] Interdisciplinary research center HALOmem, Charles Tanford Protein Center, Institute of Biochemistry and Biotechnology, Martin Luther University Halle-Wittenberg, Kurt-Mothes-Str. 3a, 06120 Halle, Germany.

[3] Department of Immunopathology, Sanquin Research, Amsterdam, Plesmanlaan 125, 1066 CX Amsterdam, The Netherlands

* Corresponding author: tampe@em.uni-frankfurt.de

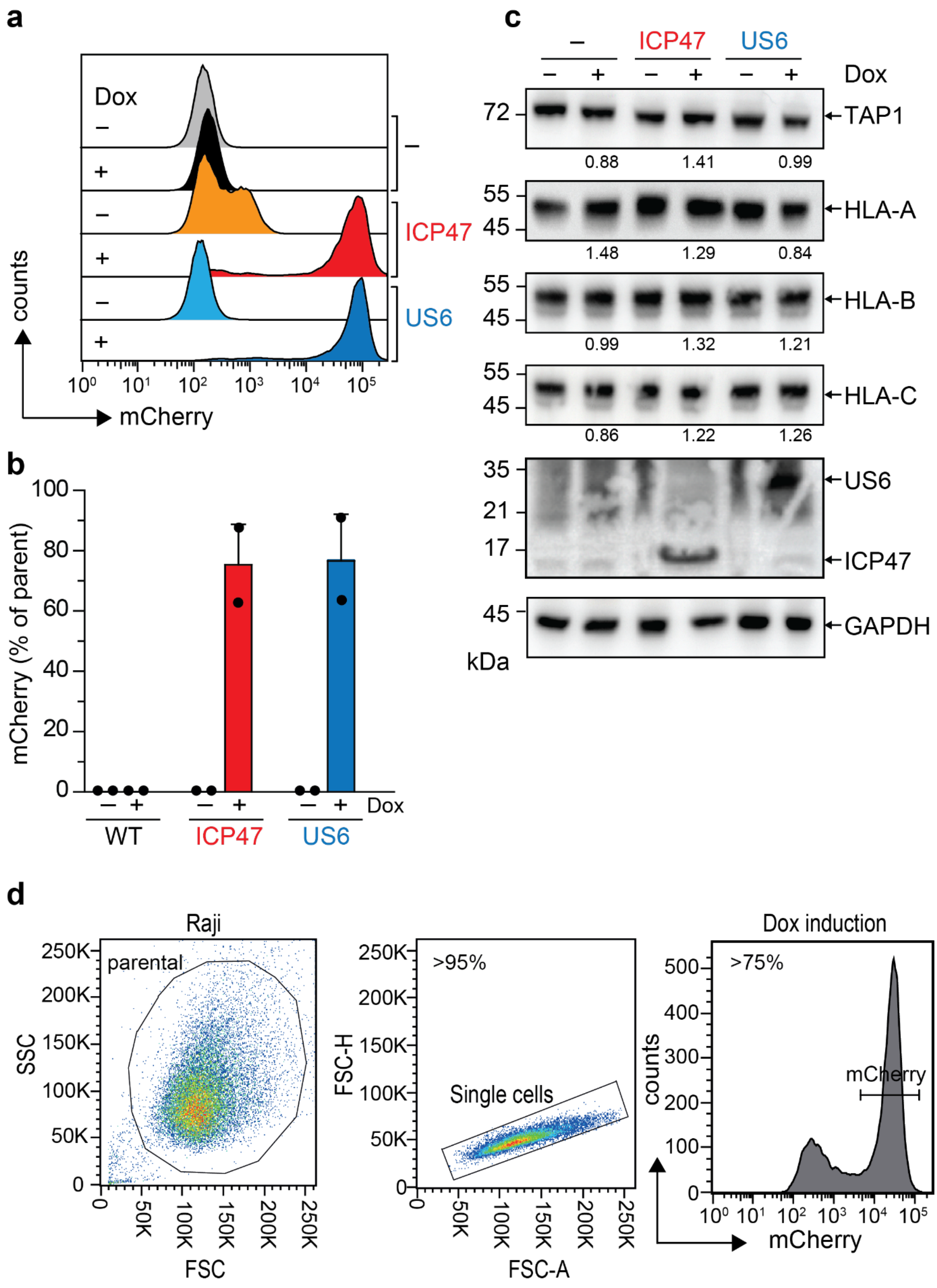

a
Dox
–
+
–
+
–
+
–
ICP47
US6
counts
10⁰ 10¹ 10² 10³ 10⁴ 10⁵
mCherry
b
mCherry (% of parent)
100
80
60
40
20
0
– + – + – + Dox
WT
ICP47
US6
c
–
ICP47
US6
– + – + – + Dox
72
TAP1
0.88 1.41 0.99
55
45
HLA-A
1.48 1.29 0.84
HLA-B
0.99 1.32 1.21
HLA-C
0.86 1.22 1.26
35
21
17
US6
ICP47
45
GAPDH
kDa
d
Raji
parental
SSC
FSC
250K
200K
150K
100K
50K
0
>95%
Single cells
FSC-H
FSC-A
Dox induction
>75%
mCherry
counts
500
400
300
200
100
0

Supplementary Figure 1. Induced expression of ICP47 and US6 in stably transduced Mel JuSo cells. **a**, Mel JuSo cells were stably transduced with ICP47- or US6-encoding lentivirus. The expression of the viral proteins was induced with 2 µg ml^{-1} doxycycline (Dox) for 16 h. Protein production was monitored by bicistronic mCherry expression via flow cytometry. The horizontal line in the histogram shows mCherry expression in ICP47 (red) and US6 (blue) encoding Mel JuSo cells and non-transfected cells (black/grey) upon doxycycline induction. **b**, Quantification of mCherry expression in ICP47- (red bar) and US6-induced cells (blue bar) in comparison to the non-induced cells (grey/back bar; n=2). **c**, Immunoblot analysis of TAP1, HLA-A, HLA-B, HLA-C, anti-SBP tag in the total cell lysate of stably transfected induced, non-induced Mel JuSo cells or wild-type Mel JuSo cells. GAPDH was used as a loading control. The quantification of densitometries of TAP1, HLA-A, HLA-B, and HLA-C normalized to the loading control GAPDH in the whole cell lysate of doxycycline-induced cells compared to non-induced cells is depicted below the immunoblots. **d**, Representative data for the general gating strategy used during the flow cytometry analysis. The cells were analyzed under yellow-green laser (561 nm) for mCherry ($\lambda_{Ex/Em}$: 587 nm/610 nm) expression. Gated cell populations are indicated by the total events analyzed in the polygon marked as the parental population. Cell aggregates from the parental population were discriminated by the forward scatter height (FSC-H) vs area (FSC-A) and gated on the single cells. The histogram analysis of the single cells shows the mCherry expression upon doxycycline induction.

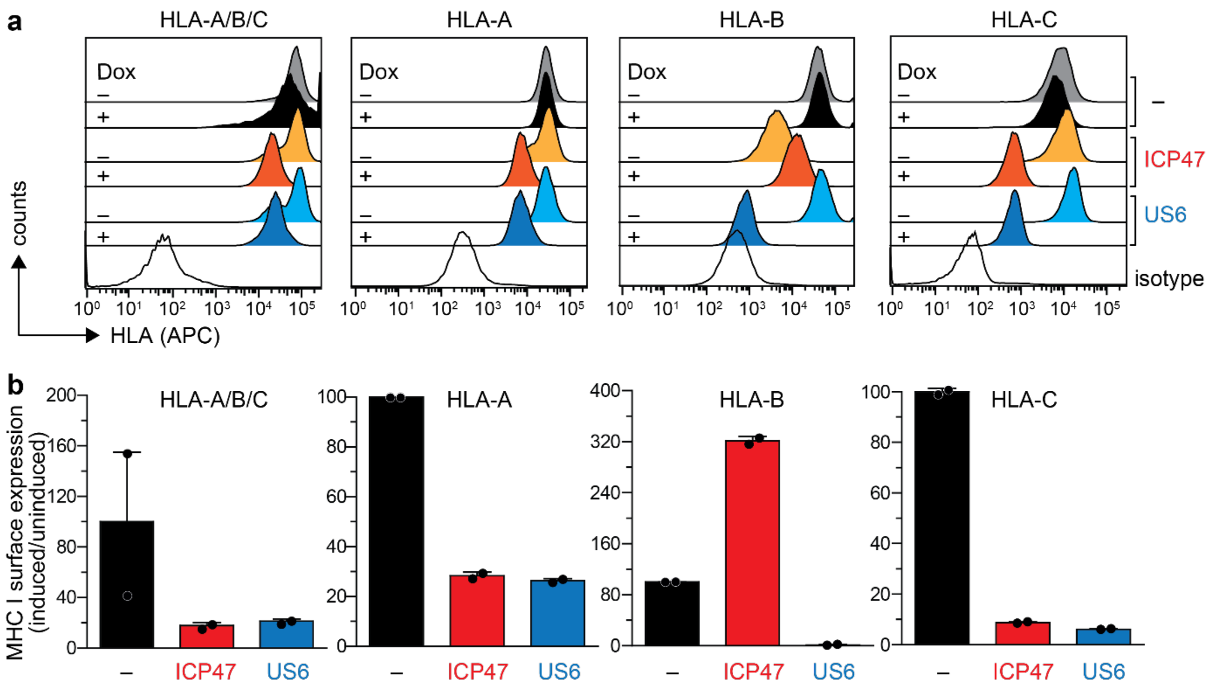

Supplementary Figure 2. Surface expression of MHC I is differentially affected by ICP47 and US6. **a**, Expression of viral factors ICP47 or US6 was induced with doxycycline in the stably transfected Mel JuSo cells. Mel JuSo express HLA-A*01:01, HLA-B*08:01, and HLA-Cw7. Surface expression of MHC I was monitored either by direct immunostaining and flow cytometry analysis using allophycocyanin (APC)-conjugated pan-HLA antibody (W6/32), or by indirect immunostaining for HLA-A (HLA-A*01:01), HLA-B (HLA-B*08:01), and HLA-C (HLA-Cw7) with primary antibody followed by APC-conjugated secondary antibody. Representative histograms showing cell surface expression of MHC I in induced and non-induced cells of wild-type cells (grey/black), ICP47-expressing (yellow/orange), and US6-expressing (blue) stable cells. The histogram with black line and white filling depicts the isotype control for the respective antibody. **b**, Quantification of the surface expression represented as the ratio of the MFI from the induced to the non-induced cells. The ratios of non-transfected cells were set as 100% (n=2). Note that the increased cell surface presentation of the HLA-B*08:01 allele upon ICP47 induction may be caused by its unique peptide-binding characteristics that lead to a high cell surface stability.

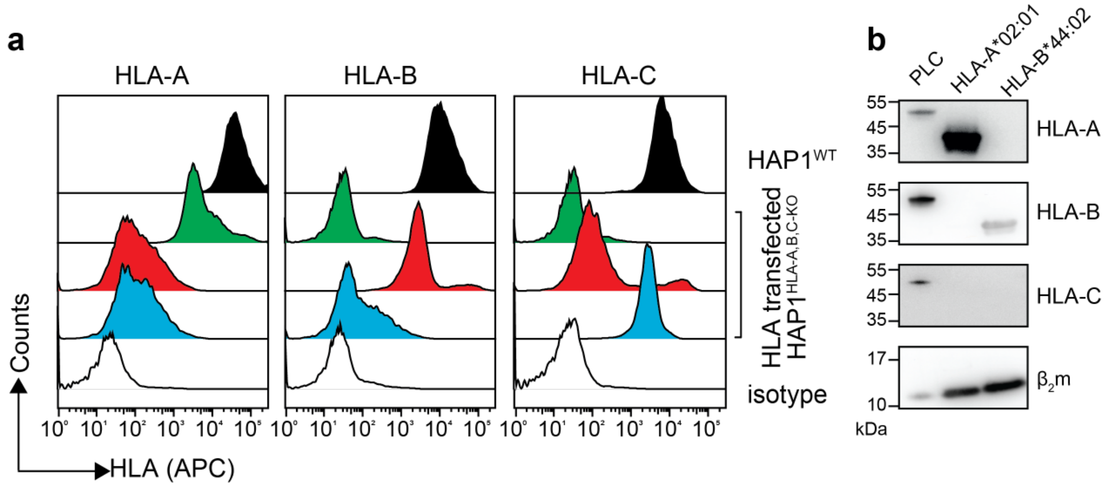

Supplementary Figure 3. **Specificity of HLA antibodies**. **a**, Flow cytometric analyses of the specificities of antibodies against allotypes HLA-A, B, and -C. Transfected cells were selected by hygromycin treatment. Wild type (WT) HAP1 cells expressing HLA-A*02:01, HLA-B*40:01, and HLA-C*03:04 were immunostained with anti-HLA-A*02, anti-HLA-B or anti-HLA-C antibodies (black histogram) as listed in Supplementary Table 1. The HLA-A,B,C knock-out HAP cells were transfected with plasmids encoding HLA-A*03:01, HLA-B*15:10, or HLA-C*04:01 and immunostained with an anti-HLA-A*03 (green histogram), an anti-HLA-B (red histogram), or an anti-HLA-C antibody (blue histogram) (Supplementary Table 1). The histogram with black line and white filling depicts the isotype control for the respective antibody. **b**, For testing the specificity of the antibodies used for WB (Supplementary Table 1), purified PLC and ectodomains of recombinant HLA-A*02:01 and HLA-B*44:02 (both lacking the transmembrane region and cytosolic tail) were analyzed by SDS-PAGE and subsequent immunoblotting using anti-HLA-A, -HLA-B, -HLA-C, and -β_2m antibodies.

Supplementary Table 1. Antibodies used for MHC I surface staining and immunoblot analysis.

Antibody	Catalogue number (Company)	Fluorescent conjugation	Purpose
TAP1	mAb148.3	-	WB
HLA-A (A*03:01)	Miltenyi (REA950)	APC	Flow cytometry
HLA-A (A*02:01)	Miltenyi (REA517)	APC	Flow cytometry
HLA-A	Abcam (Ab52922)	-	WB
HLA-B	Thermo (PA5-35345)	-	Flow cytometry
HLA-B	Abcam (Ab76795)	-	WB
HLA-C	Abcam (Ab126722)	-	Flow cytometry
HLA-C	BioLegend (373302)	-	WB
Secondary	BioLegend (405308)	APC	Flow cytometry
Secondary	Abcam (Ab130805)	APC	Flow cytometry
IgG1	BioLegend (400302)	APC	Isotype control, flow cytometry
IgG1	Abcam (Ab172730)	APC	Isotype control, flow cytometry
HLA-A/B/C (W6/32)	BioLegend	APC	Flow cytometry
β_2m	Sigma (HPA006361)	-	WB

APC – allophycocyanine; WB – Western blot

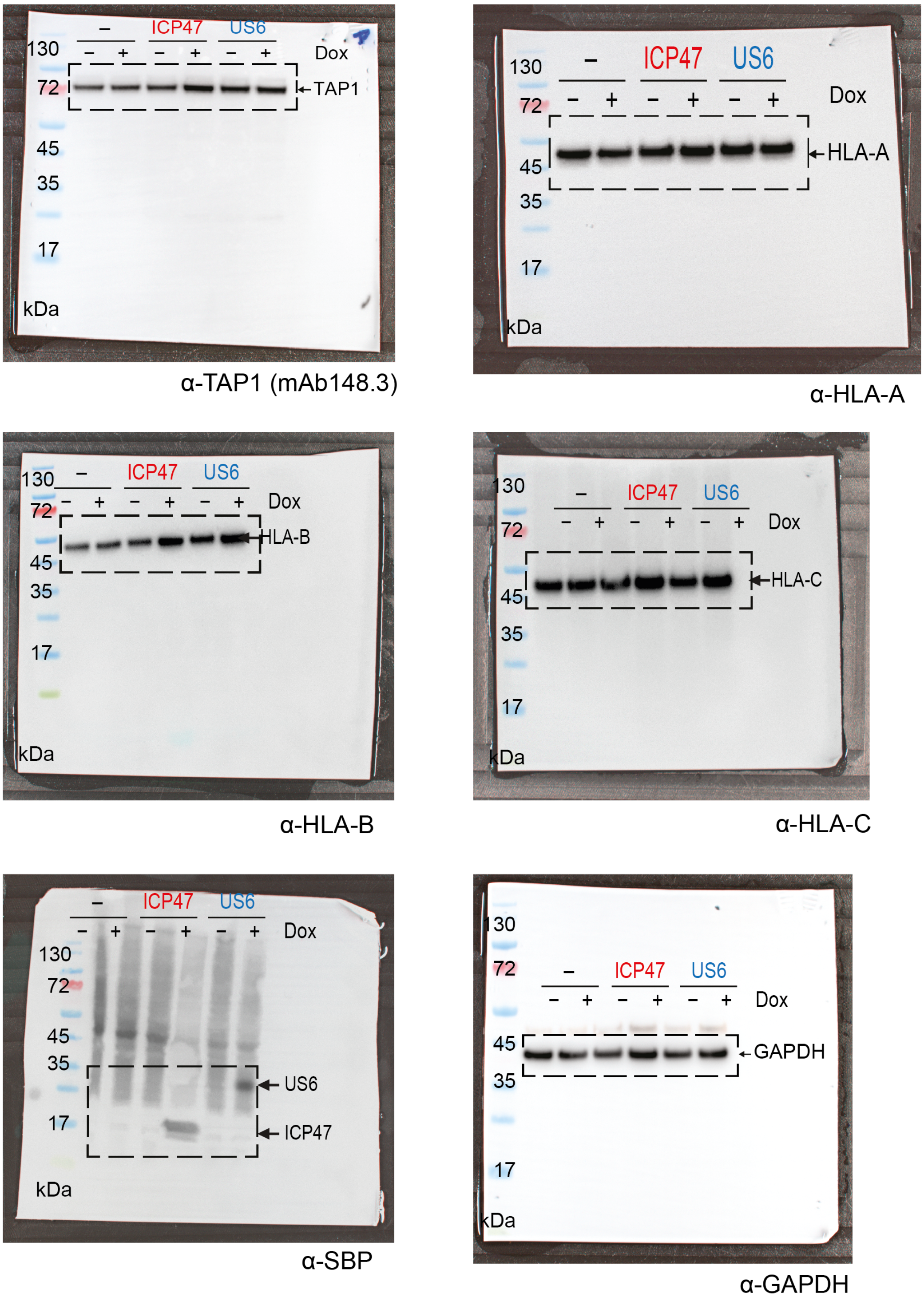

Supplementary Figure 4. Uncropped immunoblot corresponding to Fig. 1c. Immunoblot analysis of TAP1, HLA-A, HLA-B, HLA-C, and SBP-tag in total cell lysates of stably transfected non-induced (- Dox) or induced (+Dox) Raji cells, or wild-type Raji cells. GAPDH was used as a loading control.

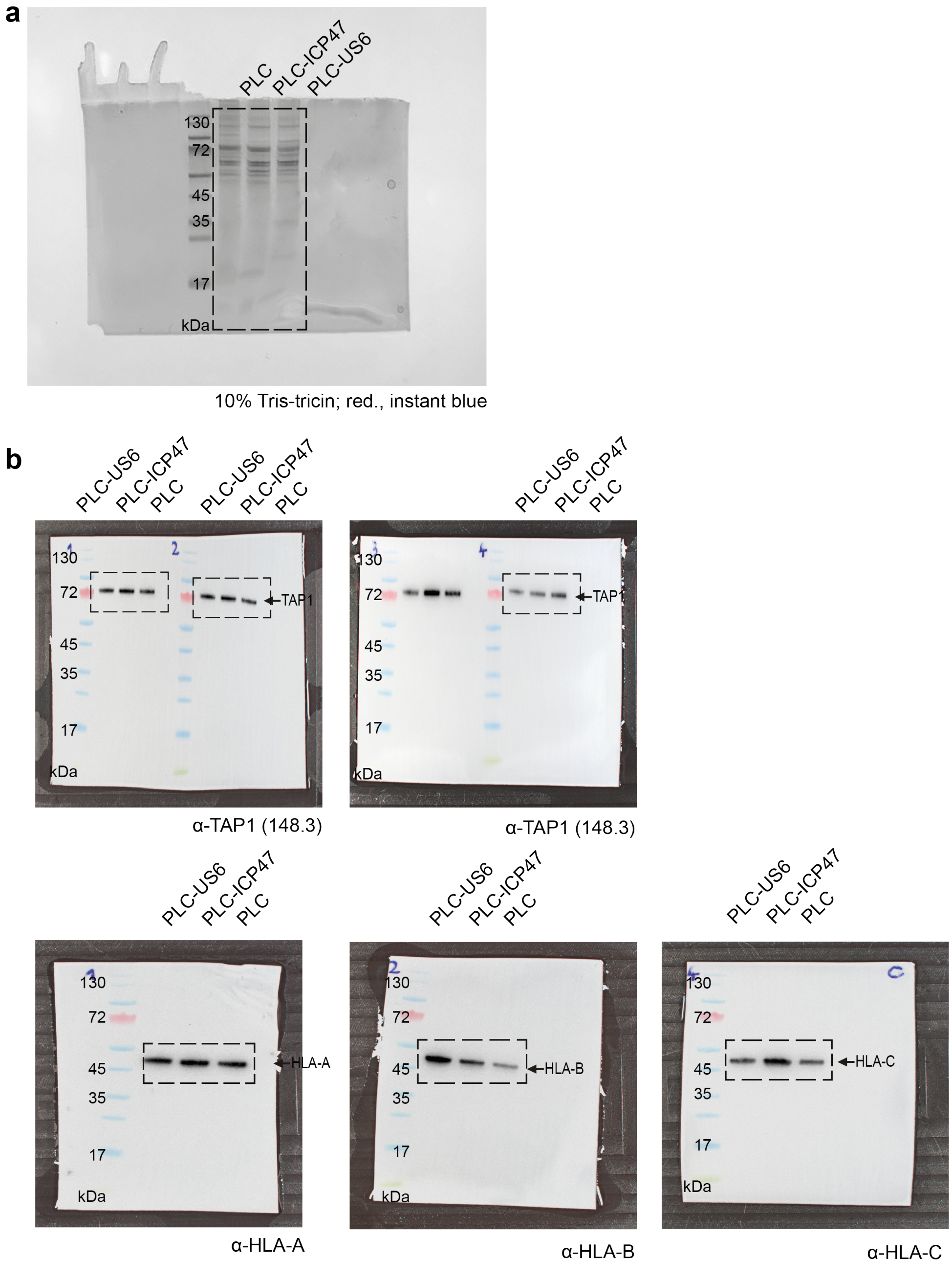

Supplementary Figure 5. Uncropped immunoblot corresponding to Fig. 4a and 4b. Viral immune evasion factors differentially change the MHC I repertoire in the PLC. (**a**) PLC was purified via the SBP-tag on ICP47 or US6 (PLC^{ICP47} or PLC^{US6}, respectively) after doxycycline induction or with a biotin-conjugated TAP1 antibody (PLC). Affinity-isolated PLCs were further purified by size-exclusion chromatography, and peak fractions were analyzed by

SDS-PAGE. (**b**) Affinity-isolated PLCs were run on SDS-PAGE and subsequent immunoblotting using HLA-A, HLA-B, HLA-C, and TAP1 specific antibodies.

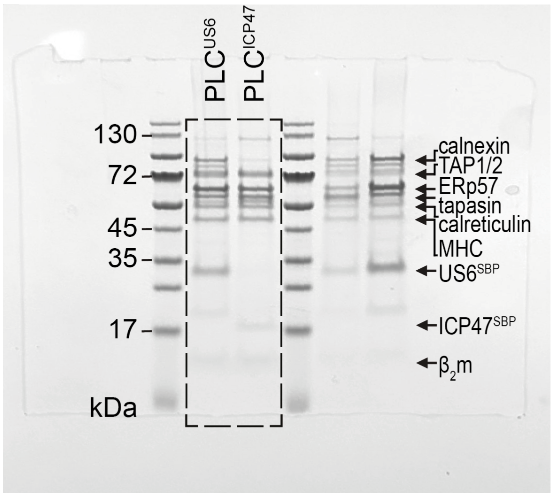

Supplementary Figure 6. Uncropped immunoblot corresponding to Fig. 5b. Intensity-based absolute quantification of the altered MHC I repertoire in ICP47- and US6-arrested PLCs. Affinity-isolated ICP47- and US6-arrested PLCs were purified via the SBP-tag were further analysed by size-exclusion chromatography. The peak fraction from the SEC were analyzed by SDS-PAGE.

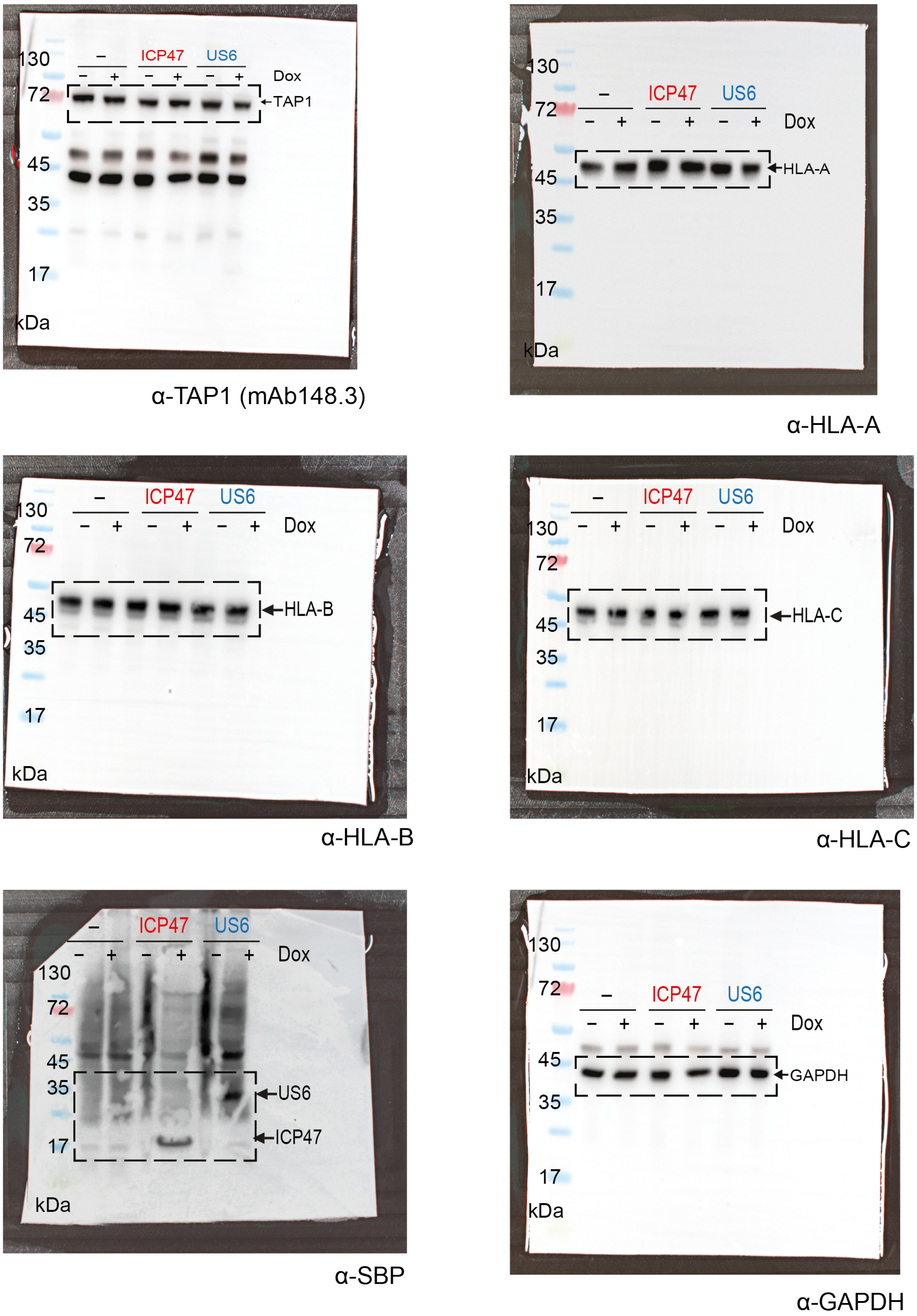

Supplementary Figure 7. Uncropped immunoblot corresponding to supplementary figure 1c. Immunoblot analysis of TAP1, HLA-A, HLA-B, HLA-C, and SBP-tag in total cell lysates of stably transfected non-induced (- Dox) or induced (+Dox) Mel JuSo cells, or wild-type Mel JuSo cells. GAPDH was used as a loading control.

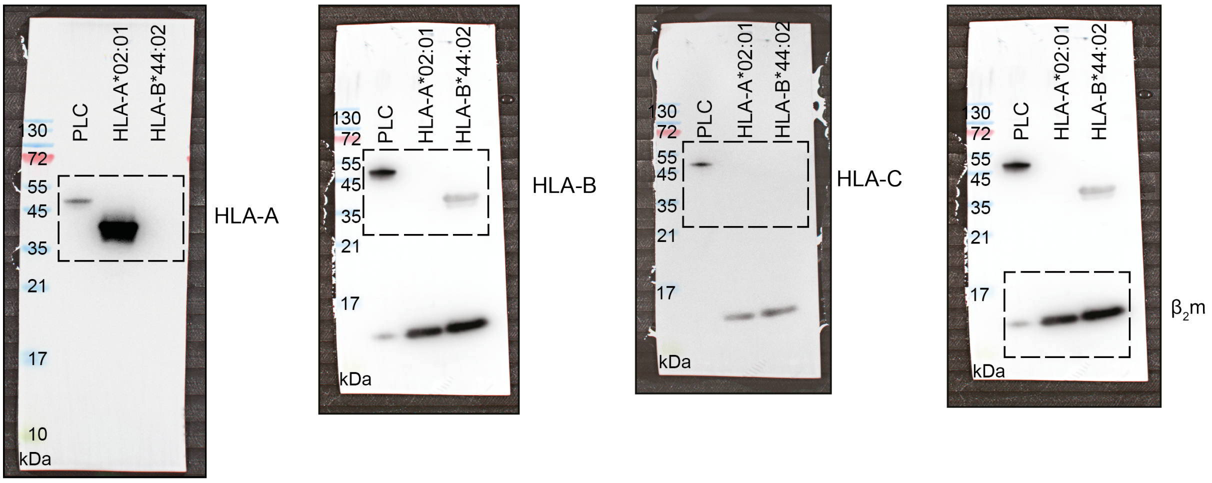

Supplementary Figure 8. Uncropped immunoblot corresponding to supplementary figure 3b. Specificity of HLA antibodies. The specificity of the antibody used for western-blot was carried out by SDS-PAGE and subsequent immunoblot analysis the purified PLC and the ectodomains of the HLA-A and HLA-B with antibodies against HLA-A, HLA-B, and HLA-C. The HLA-B and HLA-C immunoblot was then re-blotted with β_2m antibody.
